# ReactomeGSA - Efficient Multi-Omics Comparative Pathway Analysis

**DOI:** 10.1074/mcp.TIR120.002155

**Published:** 2020-09-09

**Authors:** Johannes Griss, Guilherme Viteri, Konstantinos Sidiropoulos, Vy Nguyen, Antonio Fabregat, Henning Hermjakob

**Affiliations:** 1 European Molecular Biology Laboratory, European Bioinformatics Institute (EMBL-EBI), Wellcome Genome Campus, Cambridgeshire, United Kingdom; 2 Department of Dermatology, Medical University of Vienna, Vienna, Austria

**Keywords:** Pathway analysis, data evaluation, bioinformatics software, melanoma, cancer biology*, cancer immunology, multi-omics data integration, tumor microenvironment

## Abstract

Pathway analyses are key methods to analyze 'omics experiments. Nevertheless, integrating data from different 'omics technologies and different species still requires considerable bioinformatics knowledge.

Here we present the novel ReactomeGSA resource for comparative pathway analyses of multi-omics datasets. ReactomeGSA can be used through Reactome's existing web interface and the novel ReactomeGSA R Bioconductor package with explicit support for scRNA-seq data. Data from different species is automatically mapped to a common pathway space. Public data from ExpressionAtlas and Single Cell ExpressionAtlas can be directly integrated in the analysis. ReactomeGSA greatly reduces the technical barrier for multi-omics, cross-species, comparative pathway analyses.

We used ReactomeGSA to characterize the role of B cells in anti-tumor immunity. We compared B cell rich and poor human cancer samples from five of the Cancer Genome Atlas (TCGA) transcriptomics and two of the Clinical Proteomic Tumor Analysis Consortium (CPTAC) proteomics studies. B cell-rich lung adenocarcinoma samples lacked the otherwise present activation through NFkappaB. This may be linked to the presence of a specific subset of tumor associated IgG+ plasma cells that lack NFkappaB activation in scRNA-seq data from human melanoma. This showcases how ReactomeGSA can derive novel biomedical insights by integrating large multi-omics datasets.

## Abstract (precis)

We present the novel ReactomeGSA resource for comparative pathway analyses of multi-omics datasets. ReactomeGSA is accessible through Reactome's web interface and the novel ReactomeGSA R Bioconductor package with explicit support for scRNA-seq data.

We showcase ReactomeGSA's functionality by characterizing the role of B cells in anti-tumour immunity. Combining multi-omics data of five TCGA studies reveals marked opposing effects of B cells in different cancers. This showcases how ReactomeGSA can quickly derive novel biomedical insights by integrating large multi-omics datasets.

## Graphical Abstract

**Figure:**
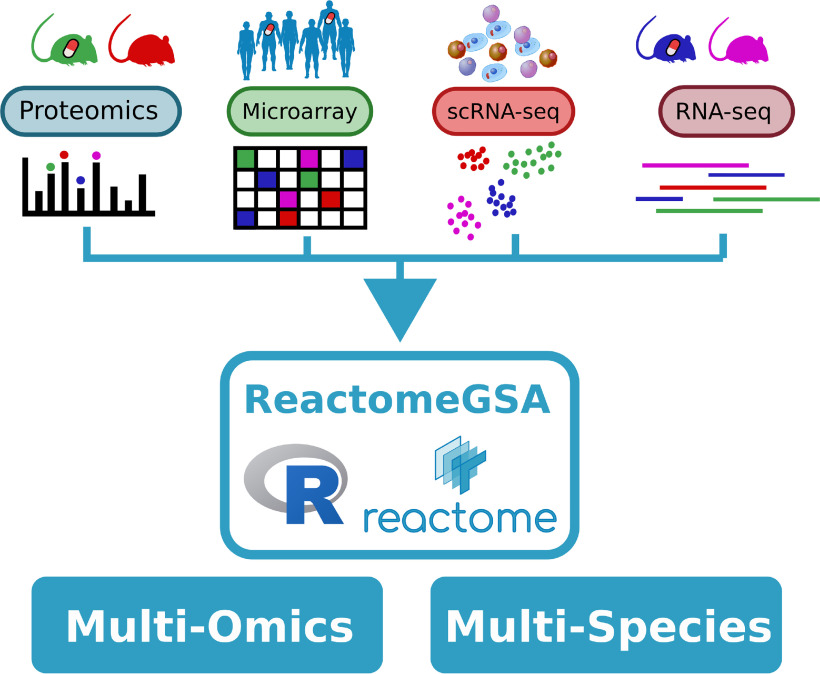

- ReactomeGSA is a novel tool for multi-species, multi-omics pathway analysis.
- Its quantitative pathway analysis methods offer high statistical power.
- Combining data of five TCGA studies shows B cells have opposing effects in cancers.
- ReactomeGSA reveals differences in key pathways between transcript- and protein-level.

Increasingly available approaches such as transcriptome sequencing (RNA-seq), MS-based shotgun proteomics, and microarray studies enable us to characterize genome- and proteome-wide expression changes. This leads to the challenge of deriving relevant biological insights from lists of hundreds of regulated genes and proteins.

Pathway analysis techniques have emerged as a solution to this problem. Resources like the Gene Ontology (GO) (1), the Kyoto Encyclopedia of Genes and Genomes (KEGG) (2), the Molecular Signatures Database (MSigDB) (3), or Reactome (4) organize existing biological knowledge into gene sets or pathways. Pathway analysis approaches can use these resources to represent long lists of regulated genes and proteins as biologically defined pathways. This leads to a more intuitive interpretation of the data and increases the statistical power. Although single genes or proteins may only show small, nonsignificant changes, synchronous changes within a pathway may reveal a biologically important effect. Thereby, pathway analysis has become an essential resource for 'omics data analyses.

The increasing availability of public 'omics datasets has made it common practice to include these into analyses. These data integration is commonly complicated if datasets were created in different species or using different 'omics approaches. Pathway analysis approaches offer a solution to this problem because data can be mapped to the more general and comparable pathway space.

Existing web-based pathway analysis resources, such as PANTHER (5), the Database for Annotation, visualization and Integrated Discovery (DAVID) (6) or Reactome's pathway analysis (7) all provide over-representation analyses. This type of pathway analysis only tests whether a list of genes is overrepresented in a specific pathway. These approaches have the advantage that the user input is simple, but ignore any underlying quantitative information at the cost of reduced statistical power. Moreover, users must manually separate up- and down-regulated genes and process them in separate analyses. Thereby, any result is only a partial representation of the underlying biological changes.

The recently developed iLINCS resource extends the concept of single-resource pathway analysis to a powerful multi-omics and multi-resource analysis (8). It tests whether a list of gene/protein identifiers correlates with a large set of pre-computed signatures. These signatures are often the result of differential expression analyses. Therefore, like the aforementioned resources, iLINCS ignores any underlying quantitative information in the final comparison. Additionally, the comparison with public data are limited to pre-defined experimental designs and comparisons whose results are stored as pre-computed signatures. Therefore, a large portion of the data remains unused.

Here, we present the novel Reactome gene set analysis system “ReactomeGSA.” ReactomeGSA supports the comparative pathway analysis of multiple independent datasets. Datasets are submitted to a single pathway analysis and represented side-by-side on the pathway level. It uses gene set analysis methods that take the quantitative information into consideration and thereby performs the differential expression analysis directly on the pathway level. Data from different species is automatically mapped to a common pathway space through Reactome's internal mapping system. All supported gene set analysis methods are optimized for different types of 'omics approaches including single cell RNA-sequencing (scRNA-seq) data. Public datasets can be directly integrated from ExpressionAtlas and Single Cell ExpressionAtlas (9). We used ReactomeGSA to show that B cell receptor signaling is surprisingly down-regulated in B cell-rich lung adenocarcinoma in contrast to four other human cancers. We could further link this to IgG+ plasma cells in scRNA-seq data. ReactomeGSA thereby provides easy access to multi-omics, cross-species, comparative pathway analysis to reveal key biological mechanisms by integrating large 'omics datasets.

## EXPERIMENTAL PROCEDURES

The ReactomeGSA analysis system is accessible through Reactome's web-based pathway browser application (https://www.reactome.org) and the “ReactomeGSA” R Bioconductor package. Both access ReactomeGSA's web-based application programming interface (API) which is also publicly accessible at https://gsa.reactome.org.

**Fig. 1.**
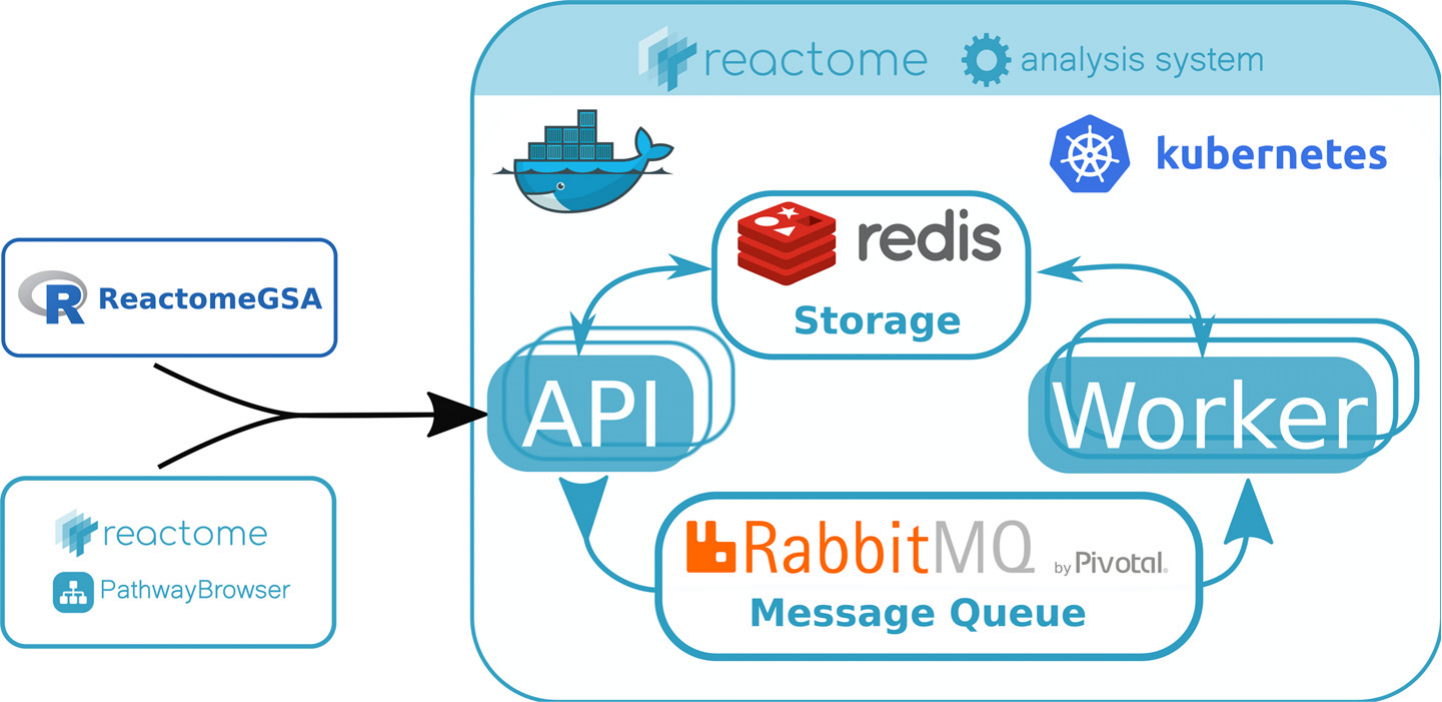
**Schema of the ReactomeGSA system.** All requests are sent to a public web-based API through the ReactomeGSA Bioconductor R package or Reactome's web-based PathwayBrowser. The system is a Kubernetes application based on the microservices architecture. All requests are distributed through an internal message queue using RabbitMQ. Worker nodes are responsible for the complete pathway analysis, including identifier mapping and the creation of the visualization data in Reactome's pathway browser. Data nodes are responsible to load data from external resources such as ExpressionAtlas. Finally, report nodes create PDF and Microsoft Excel files as a static report of the analysis results. All data are stored in a central Redis instance. All nodes are Docker containers that are orchestrated by Kubernetes and automatically scaled based on current demand. Thereby, the application can dynamically adapt to changing usage levels.

The backend is a Kubernetes application (https://kubernetes.io/) currently consisting of six deployments. Each deployment represents one Docker container (Docker Inc, https://www.docker.com). All data are stored in a Redis instance (https://redis.io/). The different components are linked through a message system provided by RabbitMQ (Pivotal, https://www.rabbitmq.com/). All components of the ReactomeGSA backend are developed in Python. The actual gene set analysis is performed using R Bioconductor (10) packages through the rpy2 (https://rpy2.github.io/) Python interface to the R language in the worker node (Fig. 1).

A key advantage of this setup is that the complete ReactomeGSA application can be described in one so-called YAML file - a Kubernetes configuration file. Because all Docker containers are freely available on Docker Hub (https://hub.docker.com) the ReactomeGSA system can be deployed using the single “kubectl apply -f reactome_gsa.yaml” command. We created a single YAML-formatted configuration file to quickly adapt ReactomeGSA to different use cases (ie. the number of resources available to the different nodes). Detailed information on how to adapt ReactomeGSA can be found on the GitHub repository (https://github.com/reactome/ReactomeGSA). Thereby, users can set up their own version of the ReactomeGSA system within minutes and deploy it locally or in the cloud.

### 

#### 

##### Multi-Omics Gene Set Analysis

At the time of writing, ReactomeGSA supports three different analysis methods: Camera through the “limma” (11) package, PADOG through the “PADOG” package (12), and the single-sample gene set enrichment analysis (ssGSEA) (13) through the “GSVA” (14) package. All pathway analyses are performed by the worker node in the ReactomeGSA system (Fig. 1).

The workflow in ReactomeGSA follows the following briefly described steps: First, the user's input data are validated in terms of experimental design, validity of submitted identifiers, and data format. Next, all identifiers are mapped to the respective human UniProt identifiers (see below). Then, the selected pathway analysis is performed for each of the submitted datasets. The parameters for the pathway analysis (such as the kernel to use for the ssGSEA analysis) is automatically chosen based on the selected data type. Finally, the pathway analysis result is converted to Reactome's internal data format to render the result in the PathwayBrowser.

Reactome's manual curation is based on human UniProt identifiers (15). Thus, as a first step in the analysis, the submitted identifiers are mapped to human UniProt using Reactome's identifier mapping system. A key issue in mapping identifiers between different identifier systems and across species is to resolve one-to-many mappings. In these cases, the ReactomeGSA system keeps an internal record of all mappings. Genes that map to multiple UniProt identifiers which all belong to the same pathway are only added once to this pathway. Thereby, one-to-many mappings are resolved at the pathway-level and inaccuracies introduced through identifier conversions are greatly reduced.

To increase the coverage of Reactome pathways, pathways can be extended through medium and high confidence interactions derived from IntAct (16). This function considerably extends Reactome's coverage.

At the time of writing, the ReactomeGSA system supports five types of quantitative 'omics data: Microarray intensities, transcriptomics raw and normalized read counts, and proteomics spectral counts and intensity-based quantitative data. Internally, these different types of data are processed using two different methods: statistics for discrete quantitative data (in case of raw transcriptomics read counts and spectral counting based quantitative proteomics data) and statistics for continuous data. For Camera and PADOG, discrete values are normalized using edgeR's (17) calcNormFactors function. Then, the data are transformed using limma's voom function (18). Continuous data are directly processed using limma (11) and normalized using limma's normalizeBetweenArrays function. The pathway analysis is subsequently performed using limma's camera function or PADOG as implemented in the respective Bioconductor R package (19). For the ssGSEA method (13) the analysis is performed using the GSVA Bioconductor R package (14). Discrete data are processed using a poisson kernel and continuous data using a gaussian kernel. Thereby, multiple types of 'omics data can be supported.

##### scRNA-Seq Pathway Analysis

The analysis of scRNA-seq data are supported through the ReactomeGSA R package's “analyze_sc_clusters” function, as well as through the direct import of data from the Single Cell Expression Atlas (9). In both cases, we calculate the mean expression of genes within a cluster. For the R package, this is done through either “Seurat”'s (20) “AverageExpression” function, or through scater's (21) “aggregateAccrossCells” function depending on the input object. Single cell data retrieved from the Single Cell Expression Atlas is processed using custom python code (see https://github.com/reactome/gsa-backend for details). This approach to create pseudo-bulk RNA-seq data resembles previously described methods to calculate differentially expressed genes (22). Thereby, all pathway analysis methods supported by the ReactomeGSA analysis system are accessible to scRNA-seq data as well.

##### TCGA B Cell Analysis

The TCGA transcriptomics data for melanoma (TCGA-SKCM) (23), lung adenocarcinoma (TCGA-LUAD) (24), lung squamous cell carcinoma (TCGA-LUSC) (25), ovarian cancer (TCGA-OV) (26), and breast cancer (TCGA-BRCA) (27) were retrieved using the “TCGAbiolinks” R Bioconductor package (28). For all datasets apart from melanoma, only primary tumor samples were retained. Genes that were expressed in less than 30% of the samples with at least 10 reads were removed.

The abundance of plasmablast-like B cells (TIPB) was quantified using the single-sample Gene Set Enrichment Analysis (ssGSEA) method (13) as implemented in the “GSVA” R Bioconductor package (14). Plasmablast-like B cells were described as CD38, CD27, and PAX5 (29). Samples were classified as TIPB-high and -low split by the median expression of the TIPB signature in all samples of the cohort. Overall survival was assessed using the R “survival” package.

The comparative pathway analysis was performed using the ReactomeGSA R Bioconductor package. In all studies, plasmablast “high” and “low” samples were compared with each other using PADOG (12).

The complete R code of this analysis, including the detailed versions of all R packages used is available in the respective Jupyter notebook (see Data availability).

##### CPTAC Data Analysis

Data processed through the common data analysis pipeline (CDA) was downloaded from the CPTAC data portal (breast cancer at https://cptac-data-portal.georgetown.edu/cptac/s/S015, ovarian cancer at https://cptac-data-portal.georgetown.edu/cptac/s/S020). For breast cancer (30), we used the proteome-level iTRAQ summary, for ovarian cancer (31) the PNNL-based protein-level iTRAQ summary. Samples were matched to the respective TCGA samples through the short barcode using the first 11 characters. Only unambiguous matches were retained. Plasmablast abundance-based groupings were transferred from the respective TCGA data set. The data were analyzed using the ReactomeGSA R package and PADOG.

##### Example scRNA-Seq Analysis

Raw read counts of the scRNA-seq data set by Jerby-Arnon *et al.* (32) were retrieved from the Gene Expression Omnibus (GEO, identifier GSE115978). The data were processed using “Seurat” version 3.1 (20) following the new scTransform normalization strategy regressing out the patient and cohort properties. To identify the B cells from the total number of cells we used the first 35 components of the principal component analysis for the subsequent steps. The neighbor graph and clustering was performed using the default parameters. B cell clusters were identified based on a high expression of CD20 (MS4A1), CD79A, CD19, and CD138 (SDC1).

B cells were extracted from the data set and re-processed, starting with the normalization step. Here, the top 11 components of the principal component analysis were used for the respective analysis steps. B cell clusters were subsequently classified following the strategy by Sanz *et al. (*33*)*. Plasmablast-like B cells and plasma cells were differentiated based on a low expression of MS4A1 (CD20) in plasmablast-like B cells. Finally, the ssGSEA analysis was performed using the ReactomeGSA R packages' analyze_sc_clusters function.

The complete workflow including the detailed versions of all used R packages can be found in the respective Jupyter notebook (see Data availability).

## RESULTS

ReactomeGSA can be accessed through Reactome's web interface (https://www.reactome.org/PathwayBrowser/#TOOL=AT) or through the novel “ReactomeGSA” R Bioconductor package (https://doi.org/doi:10.18129/B9.bioc.ReactomeGSA, Fig. 1). Both access the public API (https://gsa.reactome.org) to perform the pathway analysis. The analysis system is a Kubernetes application based on the microservice paradigm that automatically scales to current demand (see Methods for details). This infrastructure enables us to offer computationally expensive pathway analysis methods through an open interface. ReactomeGSA currently supports three methods: PADOG (12), Camera through the limma R package (11), and the ssGSEA (13) through the GSVA (14) R package (see Experimental Procedures for details). Although PADOG more often ranks biologically important pathways higher than other approaches, it is computationally more expensive. In such cases, Camera, which does not rely on permutations but linear models, results in faster results. ssGSEA is not a gene set enrichment analysis but aggregates expression values on the pathway level. This is helpful if the analyzed samples cannot be attributed to clear phenotypes or are to be correlated with continuous parameters such as survival time. The API and its complete specification is publicly available at https://gsa.reactome.org. Thereby, ReactomeGSA can easily be integrated into any other software infrastructure.

**Fig. 2.**
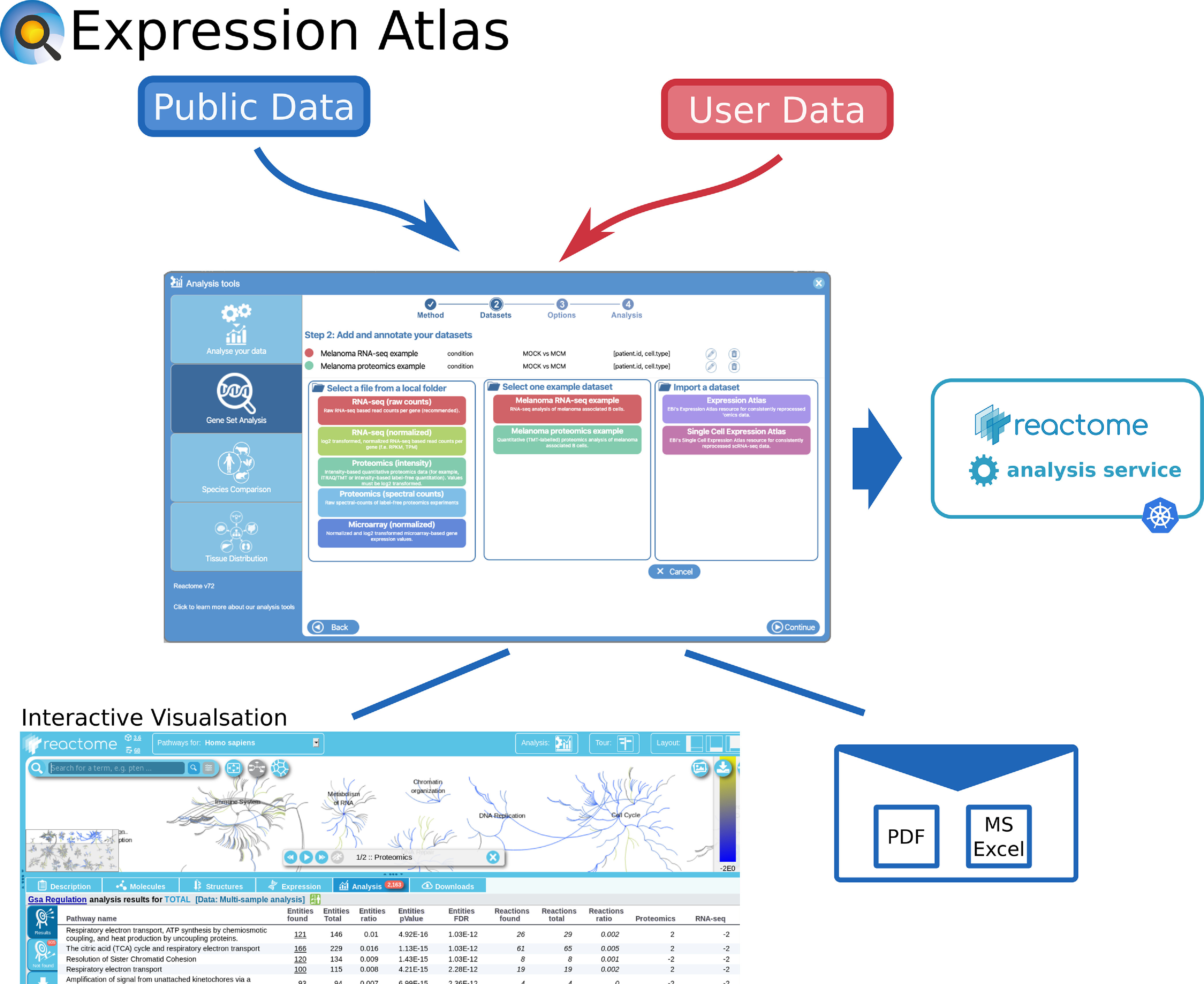
**ReactomeGSA is fully integrated into the web-based Reactome pathway browser (https://reactome.org).** Users can either upload their own datasets or import public data from ExpressionAtlas. The gene set analysis is performed through the ReactomeGSA API. Results are visualized in Reactome's interactive pathway browser and sent as static reports in PDF and Microsoft Excel format via E-mail.

ReactomeGSA is fully integrated in Reactome's existing web-based pathway browser application (Fig. 2). After choosing the new “Analyse gene expression” tab and the desired analysis method, the user can add any number of datasets to the analysis request. Public datasets are directly loaded from Expression Atlas and the Single Cell Expression Atlas (9). Results can be sent as emails including static PDF and Microsoft Excel reports. Finally, the complete gene set analysis result is visualized in Reactome's interactive pathway browser. The pathway browser enables users to view Reactome's complete pathways from a tree-based, hierarchical overview, down to the single gene- and protein-level reactions. The results of different datasets can be switched at the click of a button or automatically changed every few seconds like a slideshow across all results. Thereby, differences between the analyzed datasets are immediately visible and can subsequently be interactively investigated down to the single gene or protein level.

**Fig. 3.**
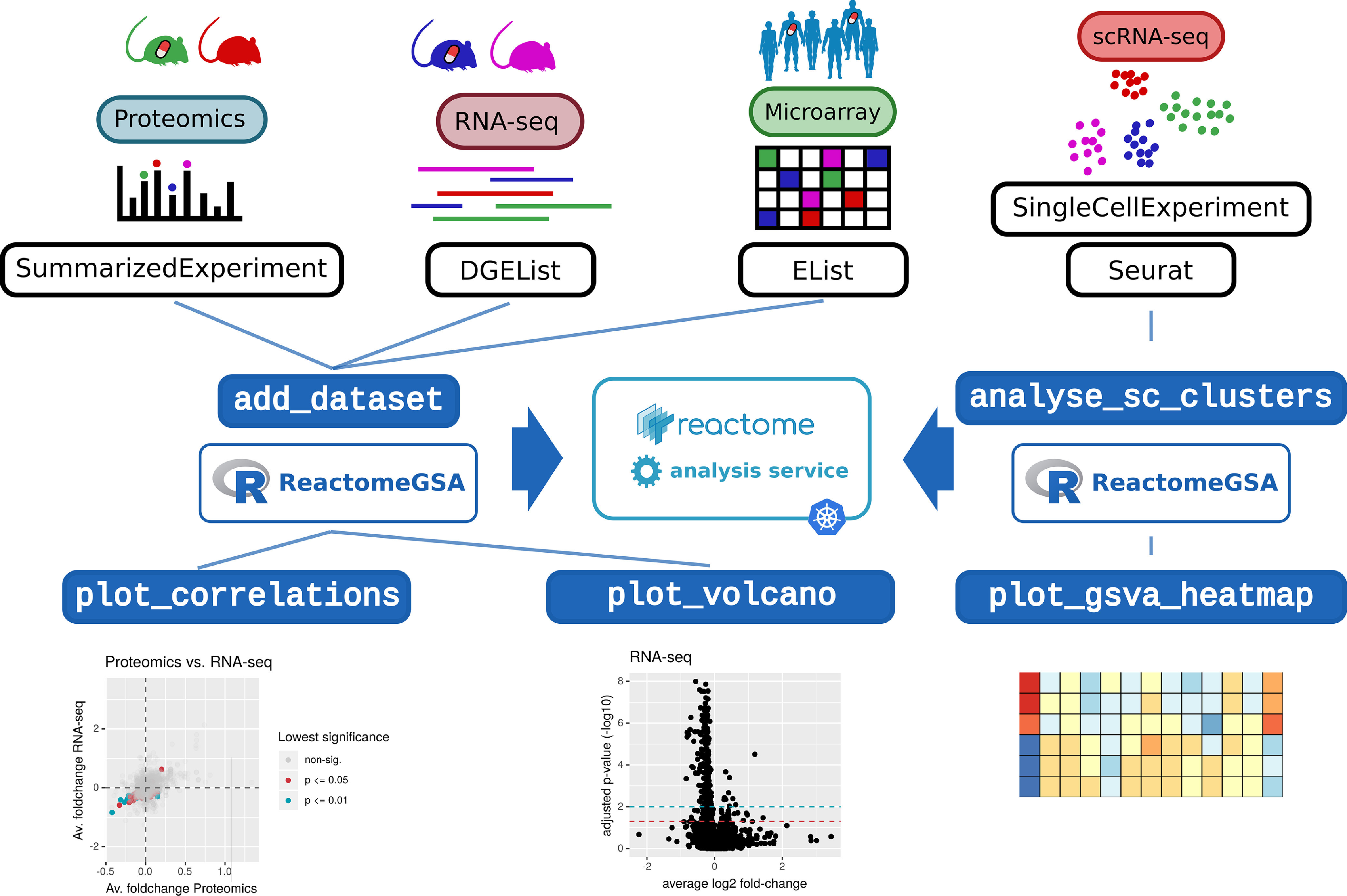
**The ReactomeGSA Bioconductor R package can directly process data from the most commonly used data structures for 'omics analyses.** The pathway analysis is performed through the ReactomeGSA analysis system and made available through a native R object. Convenient plotting functions give a quick overview of how well two datasets correlate on the pathway level. Volcano plots further highlight the magnitude of the observed changes in individual datasets. Additionally, pathway analysis of scRNA-seq data are simplified through the single “analyse_sc_clusters” function.

The ReactomeGSA R package has been included in Bioconductor since version 3.10 (Fig. 3). Like the web interface, multiple datasets can be added to a ReactomeAnalysisRequest object. Expression values and metadata can directly be loaded from Bioconductor ExpressionSet, limma EList (11) and edgeR (17) DGEList objects. Thereby, the ReactomeGSA package can easily be integrated into existing R-based workflows. The analysis results are returned as a ReactomeAnalysisResult object. This object contains the pathway analysis results across all analyzed datasets, as well as the gene- or protein-level results of the differential expression analysis. It can directly open the interactive visualization in Reactome's web-based pathway browser (see above) and create plots to visualize the comparative pathway analysis results. Thereby, the multi-data set results generated by ReactomeGSA can be natively processed in R.

The ReactomeGSA R package has dedicated features to simplify pathway analyses of scRNA-seq data (Fig. 3). The “analyse_sc_clusters” function can directly process Seurat (20) and Bioconductor's SingleCellExperiment objects (22). It automatically retrieves the average gene expression per cell cluster and performs an ssGSEA analysis on the cluster-level expression values. This results in one pathway-level expression value per cell cluster. Thereby, cell clusters can quickly be interpreted based on specific biological functions.

### 

#### 

##### ReactomeGSA Reveals a Lack of B Cell Activation in B Cell-Rich Lung Adenocarcinoma

We were among the first to show that B cells play a crucial role in anti-tumor immunity in human melanoma (29). *In vitro*, B cells differentiate toward a TIPB phenotype in the presence of melanoma cells. The corresponding molecular TIPB signature predicts overall survival in the TCGA melanoma cohort. Whether this effect is specific to melanoma or whether it is a general part of the anti-tumor immune response is currently unknown.

**Fig. 4.**
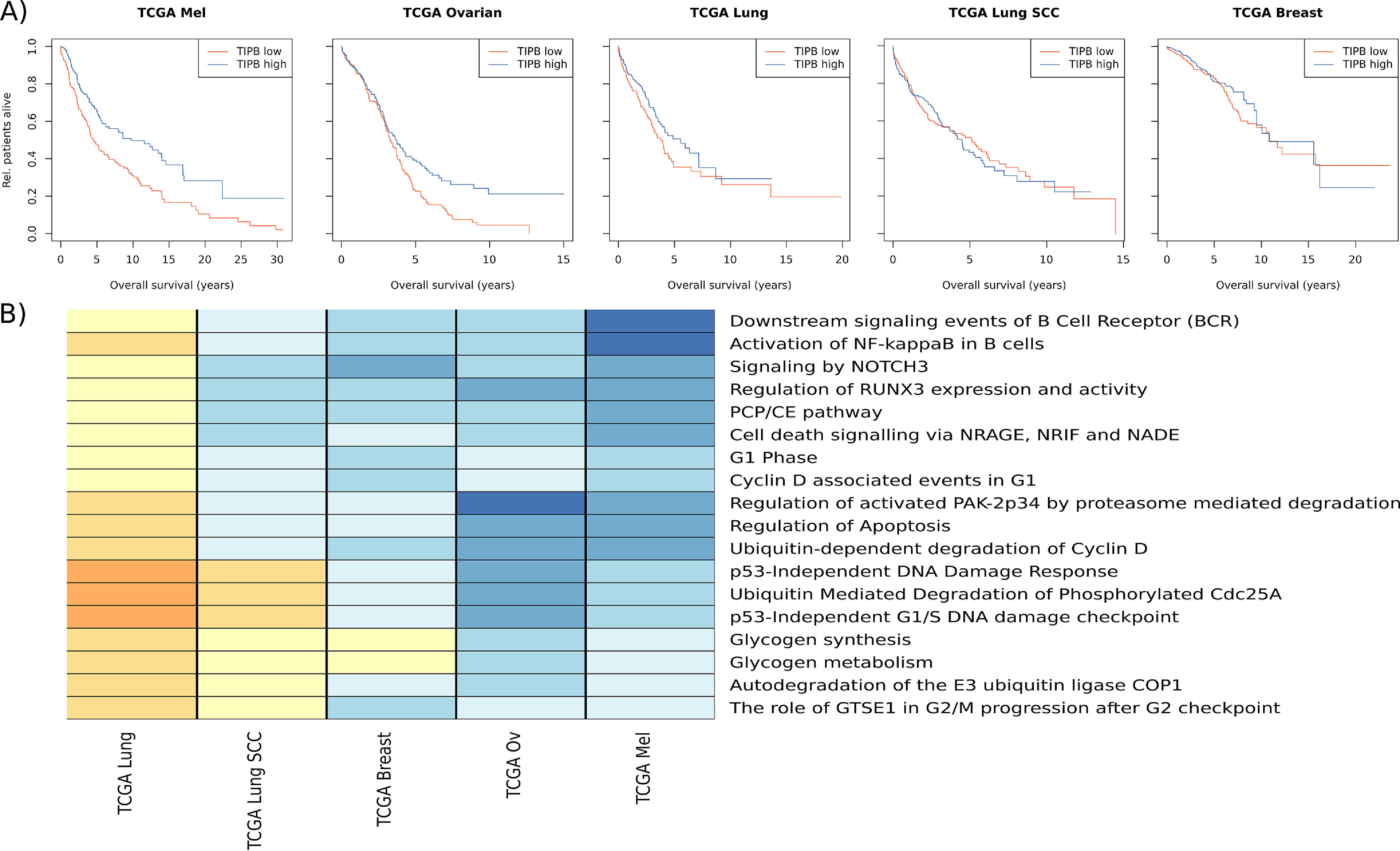
**Comparison of TIPB-high *versus* -low samples from TCGA studies on melanoma (TCGA Mel), ovarian cancer (TCGA Ovarian), lung adenocarcinoma study (TCGA Lung), lung squamous cell carcinoma (TCGA Lung SCC), and breast cancer (TCGA Breast).** *A*, Overall survival of patients with high (*blue* line) or low (*red* line) expression of the TIPB signature (split by the median expression in the data set). *B*, Average gene fold-changes per pathway. Only pathways significantly regulated (FDR < 0.1) in the TCGA melanoma and the TCGA lung adenocarcinoma cohort with a different direction of regulation in these two cohorts are shown. Shades of yellow represent a down-regulation, shades of blue an up-regulation.

We analyzed the difference between TIPB-high *versus* TIPB-low samples in the TCGA cohorts for melanoma (23), lung adenocarcinoma (24), lung squamous cell carcinoma (25), ovarian cancer (26), and breast cancer (27). Melanoma and ovarian cancer patients with high levels of TIPB showed significantly longer overall survival (likelihood ratio test *p* < 0.01 for both, hazard ratio 0.56 melanoma, 0.69 ovarian cancer, Fig. 4*A*). There was no significant difference in overall survival for lung adenocarcinoma, lung squamous cell, and breast cancer patients (likelihood ratio test *p* = 0.04, *p* = 0.2 and *p* = 0.9 respectively). Therefore, the effect of TIPB on anti-tumor immunity and patient survival differs across these types of cancers.

We subsequently assessed pathway-level differences between patients with high- and low-levels of TIPB in the five cohorts. The comparative pathway analysis was performed using our ReactomeGSA R package and the PADOG gene set enrichment analysis. 383 pathways were significantly regulated in at least one of the datasets (FDR < 0.1, supplemental Data S1). 64 of these pathways showed a differential regulation in one of the datasets compared with melanoma. We previously showed *in vitro* that NF-kappaB activation was significantly up-regulated in B cells after stimulation with melanoma conditioned medium (29). Lung adenocarcinoma samples were the only ones that showed a significant down-regulation of the “Activation of NF-kappaB in B cells” pathway (FDR = 0.08). Even though these samples have a higher number of TIPB, overall B cell activation is reduced.

We specifically assessed how the lung adenocarcinoma cohort differs from the melanoma cohort. In total, 18 pathways were significantly regulated in both the melanoma and the lung adenocarcinoma cohort (Fig. 4*B*). Next to the down-regulation of NF-kappaB related genes, there was an overall down-regulation of B cell receptor signaling, but also p53 related DNA damage response, cell cycle and apoptosis related pathways. This shows that lung adenocarcinoma samples with a high number of tumor induced plasmablast-like B cells have a distinct different signaling state compared with melanoma.

Pathways related to B cell receptor signaling and apoptosis correlate with the survival benefit observed through higher numbers of TIPB. The melanoma and ovarian cancer cohort both showed the strongest survival benefit which was linked to the strongest up-regulation of apoptosis related pathways but also B cell receptor signaling. These results highlight that ReactomeGSA's comparative pathway analysis can quickly reveal clinically relevant conserved signaling events.

##### Cancer-Relevant Pathways Differ in Proteomics and Transcriptomics Data

In our recent characterization of melanoma associated B cells, key phenotypic changes in B cells were primarily observed on the protein but not the transcriptome level. We performed a comparative pathway analysis of the two TCGA cohorts that were also analyzed by CPTAC using a global proteomics approach.

99 samples of the breast cancer CPTAC study (30) and 62 samples of the CPTAC ovarian study (31) could be directly mapped to samples from the respective TCGA study. As our TIPB signature was only validated for transcriptomics data, sample grouping into TIPB-high and -low samples was transferred from the TCGA data. The pathway analysis was performed using our ReactomeGSA R package and PADOG. 113 and 96 pathways were significantly regulated (FDR < 0.05, supplemental Data S2) in the proteomics and transcriptomics data from the breast and ovarian cancer study respectively. Out of these, 13 showed a different direction of regulation in the breast study, and one in the ovarian cancer study between proteomics and transcriptomics measurements. In breast cancer, these included VEGF signaling, EGFR signaling, and IGF1R signaling related pathways (all up-regulated in transcriptomics and down-regulated in proteomics). In ovarian cancer, FGFR signaling was significantly up-regulated in the transcriptomics but down-regulated in proteomics data. All of these pathways are linked to proliferation and are relevant pathways to tumor biology. B cell receptor signaling associated pathways were significantly up-regulated in all datasets. This highlights how ReactomeGSA can quickly reveal biologically relevant differences and similarities between 'omics datasets.

##### IgG+ Plasma Cells Show Reduced NFkappaB Activation

Specific subtypes of B cells seem to be primarily responsible for the B cell triggered anti-tumor response (29, 34–36). We therefore assessed whether the observed difference in NFkappaB activation is B cell subtype specific.

**Fig. 5.**
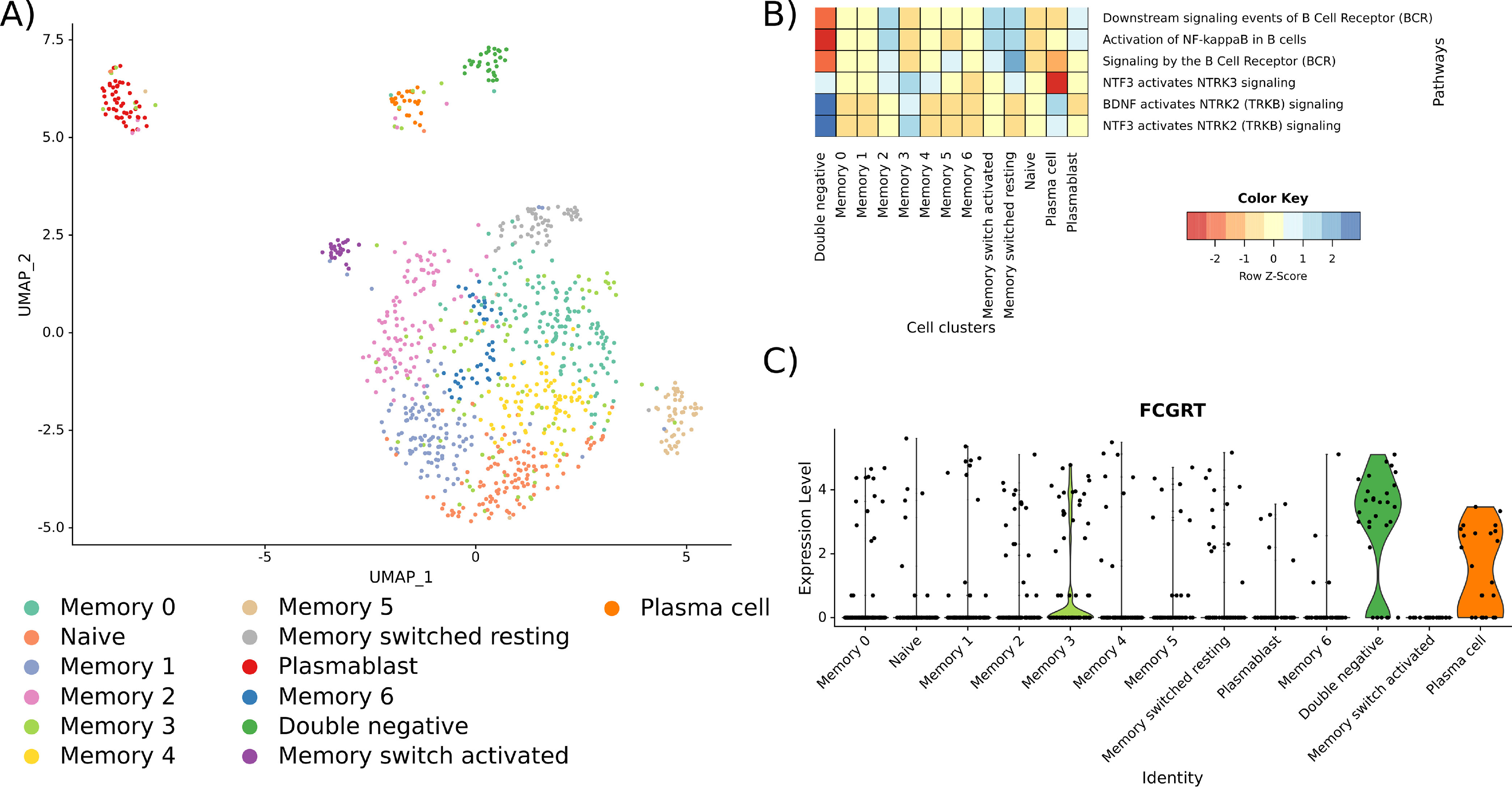
**Analysis of B cell subtypes from the data set by Jerby-Arnon *et al*. (32)** *A*, UMAP plot of the identified B cell clusters. Cell type annotations are based on canonical B cell markers (33). *B*, ReactomeGSA gene set variation based pathway-level expression in the identified B cell clusters of the Jerby-Arnon *et al.* Data set. Expression values were z-score normalized by pathway. *C*, Expression of IgG estimated through FCGRT abundance in the B cell clusters.

The extracted B cells from the scRNA-seq data set by Jerby-Arnon *et al.* (32) formed 13 distinct clusters using Seurat (see Methods for details). Based on canonical B cell markers (33) we classified these clusters as double negative B cells, seven types of memory-like B cells, memory-switched resting and -activated B cells, naive B cells, plasma cells, and plasmablast-like B cells (Fig. 5*A*). Consistent with their transitional phenotype between B cells and plasma cells, plasmablast-like B cells were the only to express SDC1 (CD138) and low levels of MS4A1 (CD20). This classification already highlights issues in classifying B cell subtypes as we had to classify seven clusters as memory B cells even though they showed marked differences in overall gene expression.

We used ReactomeGSA R package's analyse_sc_clusters function to quantify pathways in these B cell clusters. There was a considerable heterogeneity between the memory B cell clusters, as well as plasmablast and plasma cells in terms of B cell receptor signaling (Fig. 5*B*). In the latter, this matches the previously described lack of functional B cell receptors in IgG positive plasma cells (37). Consistently, plasma cells but not plasmablast-like B cells expressed high levels of IgG as determined through Fc fragment of IgG receptor and transporter (FCGRT) expression (Fig. 5*C*). Plasma cells and plasmablast-like B cells further differed in NTRK signaling which regulates cell survival, proliferation and motility (38). Our original TIPB signature is too coarse to perfectly differentiate between plasma cells and plasmablast-like B cells. Therefore, the lack of B cell receptor signaling in lung adenocarcinoma samples points toward the high abundance of IgG+ plasma cells. These were shown to be negative prognostic factors in lung adenocarcinoma (39) which may explain the reduced survival benefit of TIPB there.

## DISCUSSION

ReactomeGSA greatly decreases the technical challenge to perform pathway analyses of unrelated datasets irrespective of 'omics technology and investigated species. The iLINCS resource (8) is comparable in terms of the integration of different 'omics data types and public datasets. In contrast to iLINCS, ReactomeGSA does not rely on pre-computed signatures for public datasets. This limits the number of public datasets that can be integrated into a single analysis. At the same time, it gives the researcher complete freedom in terms of experimental design and data analysis strategy to use. Our analysis of TCGA datasets based on a custom signature, for example, would not be supported by iLINCS. Additionally, ReactomeGSA directly supports quantitative 'omics data as input. Thereby, we can use gene set analysis approaches with increased statistical power compared with simple overrepresentation analysis (19). The support for sample-level quantitative data enables us to integrate gene set variation analyses which we found especially helpful in the analysis of scRNA-seq data. We, thus, believe that the ReactomeGSA system is a considerable step forward in giving researchers easy access to complex, more sophisticated pathway analysis methods.

Nevertheless, ReactomeGSA is still limited to three “classic” 'omics technologies. Future plans involve supporting methods such as chromatin accessibility sequencing data. Internally, ReactomeGSA is already designed to handle different types of quantitative data. ReactomeGSA thus provides an infrastructure that is well suited to cover a large variety of 'omics technologies.

A key decision in multi-omics pathway analyses is how to integrate different types of 'omics data. Methods such as the Gene Set Omic Analysis (GSOA) (40) or the PAthway Recognition Algorithm using Data Integration on Genomic Models (PARADIGM) (41, 42) merge different 'omics measurements into a single result. Thereby, only data from the same or highly similar samples can be integrated. Moreover, differences between the different 'omics measurements disappear. As highlighted in our example data and previous studies, such differences are to be expected (29, 30). We deliberately developed a system that can highlight such differences that researchers can interactively investigate with the Reactome pathway browser. Moreover, the user can quickly choose between different pathway analysis algorithms that all have different strengths and weaknesses (43). ReactomeGSA provides a novel multi-omics pathway analysis infrastructure that is tailored to expert bioinformaticians and nonexperts alike.

## DATA AVAILABILITY

The complete source code of the ReactomeGSA backend, the web-based pathway browser, and the ReactomeGSA Bioconductor R package are available under a permissive open source license on GitHub (https://github.com/reactome). All docker images of the ReactomeGSA analysis system are publically available on Docker Hub (https://hub.docker.com). Central links to all components of the ReactomeGSA system can be found at https://reactome.github.io/ReactomeGSA. The source code of the backend (ie. the Kubernetes application) can be found at https://github.com/reactome/gsa-backend. The source code of the R package is available at https://github.com/reactome/ReactomeGSA. Additionally, a detailed documentation on how to set up the ReactomeGSA analysis system on a local Kubernetes instance can be found on https://reactome.github.io/ReactomeGSA.

The detailed API specification of the ReactomeGSA system is available on https://gsa.reactome.org. Therefore, the complete analysis capabilities can easily be integrated into any other existing software platform.

The code to analyze the example datasets presented in this manuscript can be found as Jupyter notebooks on https://github.com/Reactome/ReactomeGSA-tutorials.

## Supplementary Material

Supplementary Data 1

Supplementary Data 2
